# Occurrence dataset of reptiles and amphibians from two old-growth forest localities along the Las Piedras River, Tambopata Province, Peru

**DOI:** 10.3897/BDJ.13.e154136

**Published:** 2025-11-06

**Authors:** Brian Crnobrna, Patrick S. Champagne, Harry Fonseca Williams, Harry Turner, Grober Panduro Pisco

**Affiliations:** 1 Universidad Nacional de Ucayali, Pucallpa, Peru Universidad Nacional de Ucayali Pucallpa Peru; 2 Negocios Amazonicos Sustentables EIRL, Pucallpa, Peru Negocios Amazonicos Sustentables EIRL Pucallpa Peru; 3 Acadia University, Wolfville, Canada Acadia University Wolfville Canada; 4 University of Queensland, Brisbane, Australia University of Queensland Brisbane Australia; 5 University of Oxford, Oxford, United Kingdom University of Oxford Oxford United Kingdom; 6 Independent Researcher, London, United Kingdom Independent Researcher London United Kingdom

**Keywords:** anura, Amazon rainforest, biogeography, Crocodilia, herpetofauna diversity, Squamata, Testudines

## Abstract

**Background:**

This study presents the first watershed-wide checklist and geo-referenced dataset of amphibians and reptiles from two primary forest localities along the Las Piedras River, Madre de Dios, Peru. Compiled from pitfall traps, quadrats, transects and opportunistic observations between 2004 and 2024, the dataset contains 2,327 records across 165 species, including several new distribution records from the central basin near the Huascar River. The Madre de Dios region in south-eastern Peru is renowned for its biodiversity and old-growth rainforests, hosting diverse flora and fauna. Protected areas like the Tambopata National Reserve and Manu National Park are vital refuges for wildlife and research hubs. The region faces threats from deforestation and illegal mining, necessitating urgent conservation efforts. Despite being one of the most diverse regions for herpetofauna globally, biogeography reports from the Las Piedras River are limited. Notably, sixty non-volant mammal species and 144 fish species have been documented, along with 59 frog and 11 reptile species at the Las Piedras Biodiversity Station (LPBS). However, a comprehensive review of reptile diversity in the watershed is lacking. This study presents a survey and occurrence dataset for reptile and amphibian species at LPBS and the Amazon Research and Conservation Centre (ARCC), including opportunistic records to provide complete taxonomic coverage. Furthermore, we review and compile other reported occurrences. This dataset and review offer detailed species and geographical information, supporting further research on herpetofauna biogeography and ecology and aiding conservation efforts on the Las Piedras River.

**New information:**

This list of reptile and amphibian species from the Las Piedras River in Peru includes new records from the basin's central area, near the Huascar River's confluence. It unifies data from early efforts to find herpetofauna at the Las Piedras Biodiversity Station spanning more than ten years. Over a decade of sampling, combined with opportunistic records, comprehensive taxonomic coverage of herpetofauna on the tributary has been provided. Our dataset contains 2,327 distinct geo-referenced records, categorised into Anura (1788), Crocodilia (10), Gymnophiona (1), Squamata (517) and Testudines (11). These records span 165 identified species, along with one entry recorded at the genus level (Chironius). This dataset was structured and managed using Microsoft Excel, where geo-referenced species occurrence data were organised into standardised formats compatible with GBIF publishing requirements. The dataset was subsequently validated and formatted as a Darwin Core Archive (DwC-A), the standard format for biodiversity data sharing, using GBIF's Integrated Publishing Toolkit (IPT). This structured approach ensures interoperability and compliance with global biodiversity informatics standards, supporting its integration into herpetofauna biogeography and conservation efforts. This dataset also includes new records from the central basin of the Las Piedras River near the Huascar River confluence. By offering 2,327 distinct geo-referenced records, this dataset (https://doi.org/10.15468/sa8m3q) supports ongoing research into herpetofauna biogeography and conservation efforts in a region under increasing pressure from deforestation and other human activities.

Based on our dataset and an accompanying review of historical records and publications, we document a total of 175 herpetofauna species in the Las Piedras River watershed. This total includes 96 reptile species (ARCC = 70, LPBS = 76) and 79 amphibian species (ARCC = 64, LPBS = 69), from both geo-referenced and literature-confirmed sources.

## Introduction

Species occurrence data, which records the presence of specific species at particular locations and times, is crucial for biodiversity conservation and ecological research. These data help scientists and conservationists understand the distribution patterns, habitat preferences and population dynamics of species (Gascon et al. 2000, Gaston 2000, Swenson et al. 2012b, Jenkins et al. 2013, Souza et al. 2013). By analysing species occurrence data, researchers can identify areas of high biodiversity, track changes in species distributions due to environmental changes or human activities and prioritise areas for conservation efforts (Hoffmann et al. 2010). Moreover, these data support the development of ecological models and provide information for policy decisions aimed at preserving ecosystems and their inhabitants (Pitman et al. 2021). Ultimately, species occurrence data are a foundational resource for protecting biodiversity and maintaining ecological balance.

The Madre de Dios Region, located in south-eastern Peru, is renowned for its biodiversity and old-growth rainforests (Bernier et al. 2017). The region lies at the interface of two of the most biologically rich and geologically significant regions in the world - the Andes Mountains and the Amazon Basin. This ecotone supports exceptionally high biodiversity and complex species turnover along elevational and climatic gradients (Gentry 1993, Householder et al. 2012, Sánchez-Cuervo et al. 2020). The broader Andes-Amazon Region is a key driver of South American biodiversity, where geological uplift and climate dynamics have shaped the diversification of many lineages (Antonelli et al. 2009, Hoorn et al. 2010). Within this broader context, Madre de Dios serves as a critical lowland node in regional biogeographical patterns, characterised by intact forests, dynamic river systems and a mosaic of habitats that support diverse herpetofauna assemblages (Gentry 1993, Catenazzi et al. 2013). This region is occupied by a variety of flora and fauna, including numerous species of mammals, birds, reptiles, amphibians and insects (Erwin 1985, Gentry 1993, Pitman et al. 2011). Key protected areas within Madre de Dios, such as the Tambopata National Reserve and Manu National Park, serve as vital refuges for wildlife and are hotspots for ecological research and ecotourism (Rodriguez et al. 1994, Morales et al. 1996, Vuohelainen et al. 2012). The region's rivers, including the Madre de Dios, are lifelines that support rich ecosystems and indigenous communities of the Matchiguenga, Yine and Ese Eja ethnicities (Serrano-Rojas et al. 2022, Bennett et al. 2023). Despite its ecological significance, Madre De Dios faces challenges from deforestation, illegal mining and other human activities (Asner and Tupayachi 2017), making conservation efforts crucial for preserving its natural wealth and ecological integrity.

Within this region, Manu National Park, one of the largest protected areas in Peru, boasts at least 155 species of reptiles and 132 species of amphibians, representing some of the highest known richness values for any single Amazonian protected area (Catenazzi et al. 2013). Some research sites (e.g. Cocha Cashu, Estacion Biologica Los Amigos) within the region have had decades of herpetology sampling, providing a wealth of data on the diversity and distribution of these species. This long-term research has contributed significantly to our understanding of herpetofauna diversity and the ecological dynamics of the region (Rodriguez et al. 1990, Rodriguez et al. 1994, Doan and Arriaga 2002, Rodrigues et al. 2004, Duellman 2005.

Although the Las Piedras River is the longest tributary of the Madre de Dios River, biogeography reports from this river remain scarce for many taxa. At least sixty non-volant mammal species (excluding bats and small rodents) have been observed around the confluence of the Las Piedras and Huascar Rivers (Payne et al. 2024). Additionally, 144 species of fish have been found in the Las Piedras River and its sub-tributaries (Carvalho et al. 2012). Regarding herpetofauna, von May et al. (2009) provided the most extensive report of species occurrence, detailing anuran diversity at the Las Piedras Biodiversity Station (LPBS). Sixty species of frogs and eleven reptiles are confirmed to occur specifically on the LPBS property von May et al. 2009, Champagne et al. 2015, Turrell et al. 2016, Chavez et al. 2019, Champagne et al. 2021, Champagne 2022, Champagne et al. 2023, Champagne et al. 2024. However, a comprehensive review including reptile diversity on the property and the Las Piedras River watershed overall has not been published.

Here, we conducted a survey and reported an occurrence dataset for reptile and amphibian species at LPBS and another research site, the Amazon Research and Conservation Centre (ARCC). Additionally, we include on our species list opportunistic records from the station properties, aiming to create a more complete picture of the taxa composition. Our dataset contains detailed species and geographical information, providing comprehensive data for further research on herpetofauna biogeography and ecology.

## Sampling methods

### Sampling description

We aggregated results from three primary herpetofauna survey methods: pitfall traps, quadrats and transects - employed over varied sampling periods from 2004 to 2023. Additionally, we included opportunistic records of species that were not detected using these structured methods. The amphibian taxonomy used follows Moravec et al. (2009), Jungfer et al. (2010), Jungfer et al. (2013), Caminer and Ron (2014), Caminer et al. (2017), Rivadeneira et al. (2018), Fouquet et al. (2021), Melo-Sampaio (2023)and Frost et al. (2025). Reptile taxonomy follows Uetz et al. (2025). We accomplished species ID mainly by examination of live specimens and photos (S1). We deposited voucher specimens in the CORBIDI collection. These vouchers bear “FF” (Fauna Forever) field tags numbered FF3320-FF3724. Photographs for most species can be found at this resource (10.6084/m9.figshare.30017893).

Sampling occurred in old-growth forests at two research sites, minimum bounding geometry of each site suggests spatial coverage of ~ 589 ha at the Las Piedras Biodiversity Station (LPBS) and ~ 650 ha at the Amazon Research and Conservation Centre (ARCC). The total area sampled on the tributary, including the two sites and opportunistic observations was 25056 ha. At LPBS, structured sampling occurred along six distributed forest plots representing major habitat types, including terra firme, palm swamp and floodplain transition zones. At ARCC, survey methods were applied across trail systems and plots encompassing seasonally flooded forests, upland terraces and oxbow lake margins. These efforts ensured representative spatial coverage across each site’s primary habitats. We define old growth as forest ecosystems composed of native species in which there are very few visible indications of human activity and where ecological processes are not significantly disturbed. Both sampling localities are situated in old growth Amazonian rainforest with a minimal history of logging or land conversion and exhibit intact canopy structure, diverse vertical stratification and undisturbed forest floor composition (Bennett et al. 2023).

Table 1 contains effort values for each of the following methods employed. Transect and quadrat data for 2004 were collected in three phases between early-July and early-December. All opportunistic data from these phases were collected from photographic evidence curated by Fauna Forever, bearing metadata within the dates entered for transect and quadrat data. Phases from May 2012 to June 2014 were continuous at least every other month, in which all pitfall trapping took place.

**Pitfall Traps**

Pitfall trap arrays were designed with straight driftline fences made of plastic sheeting measuring 30 m long, trenched into soil at the bottom edge and installed with four 60 l buckets. The buckets had holes drilled in the bottom to allow drainage and were spaced 10 m apart (the second and third buckets excluded opposing sides of the driftline), although traps at ARCC employed a mixture of 20 l, 60 l and 120 l buckets in an experimental array. In all cases, buckets were left uncovered, but natural cover was provided inside the buckets to accommodate captured animals. We placed traps at opportunistic locations 200, 500 and 600 m from the base lodges. Traps were checked once every 24 hours. All individuals of all amphibian and reptile species were recorded and released at a predetermined release site approximately 100 m away from the trap.

**Quadrats**

Quadrat data originating from 2004 can be considered identical to those detailed by Doan (2003); utilising a “corner-in” method on 100 m^2^ and 400 m^2^ quadrats from two plots run systematically. Newer quadrats quadrupled the plot size and combined random walks with the “corner-in” method. These quadrats were selected randomly from the trails and large transect plots used at LPBS.

**Transects**

Non-invasive visual transect searches followed straight lines 100 m long in homogeneous forest devoid of permanent waterbodies or drastic shifts in relief (ridges, stream banks etc.). Search time was limited to less than 50 minutes measured with a stopwatch to factor out specimen processing or any time spent off transect. Sampling took place during both day and night-time and effort was made to perform three diurnal transects for every five nocturnal transects. Nocturnal transects took place between 17:30 h and 00:00 h and diurnal between 08:30 h and 13:00 h, but both took place on the same plots, numbering six at LPBS and two at ARCC. In 2004 at LPBS, two plots of 24 transects were run systematically. In 2012 and 2013, two plots at ARCC contained 10 separate transects run via a stratified-random post hoc resampling design. At LPBS in 2014, three more plots were run in random order over a much larger geographic scale. The average width of transects were 4 m.

**Opportunistic sampling**

All opportunistic encounters, valued as presence/absence data, were recorded. Time-constrained searches restricted to trails between 1.5 and 5 km in length, swamp searches and stream runs made up the opportunistic samples.

## Geographic coverage

### Description

**Figure 1.**
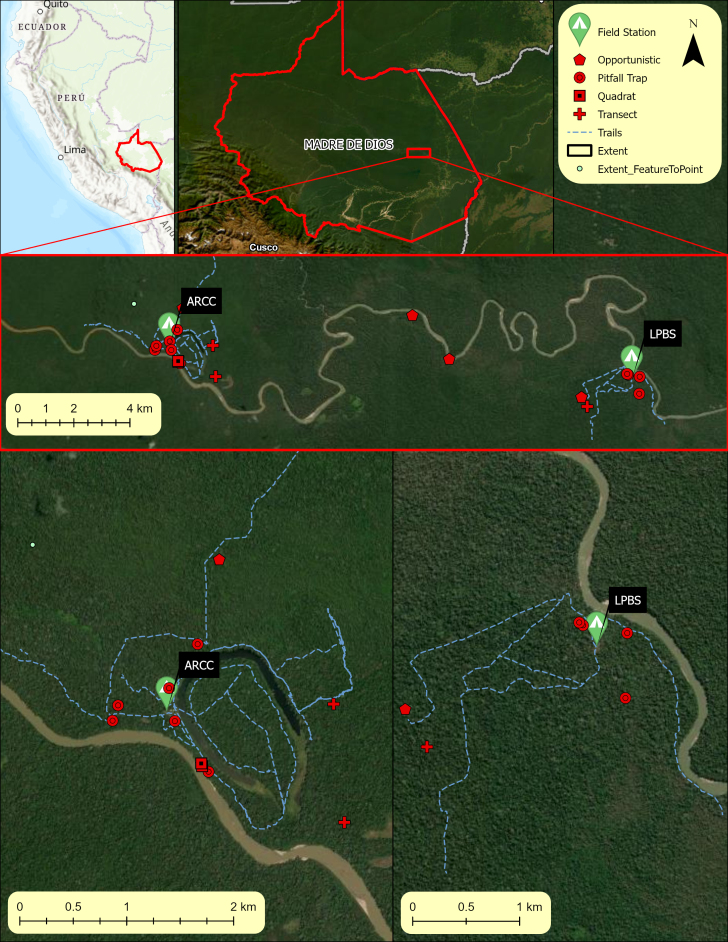
The location of two research sites on the Las Piedras River, Madre de Dios, Peru. Spatial Reference Name: Peru96 UTM Zone 19S, PCS: Peru96 UTM Zone 19S, GCS: GCS Peru96, Datum: Peru96, Projection: Transverse Mercator. Basemap imagery sourced through Esri imagery services and includes TomTom, Garmin, FAO, NOAA, USGS, Earthstar Geographics, Esri, USGS and Maxar sources. Trails and stream tracks acquired using Garmen 64s GPS.

The Amazonian Department of Madre de Dios in south-eastern Peru is named after the largest river present, the Madre de Dios River. The Department is situated north of Puno, east of Cusco and south of Purús Province and covers roughly 85,300 km^2^. The capital city of Puerto Maldonado is located at the confluence of the Tambopata and Madre de Dios Rivers. The Las Piedras River is the longest tributary of the Madre de Dios River in Peru and the 14^th^ longest river in Peru, with a length of 640 km. The river drains into the Madre de Dios, approximately 10 km northwest of Puerto Maldonado and originates from highlands in Alto Purús National Park. Our study area covers a stretch of 65 km of the Las Piedras River near the confluence of the Huascar River. The two study sites, the Las Piedras Biodiversity Station (LPBS) and the Amazon Research and Conservation Centre (ARCC), are located in the central area of the Las Piedras River tributary (Fig. 1). The two sites are surrounded by several larger and sustainably managed Brazil nut, ecotourism and conservation concessions (approximately 20,000 hectares) (Payne et al. 2024). The habitat is consistent with the typical characteristics of the lowland Amazon rainforest under 500 m in the Madre de Dios Region.

LPBS is a privately protected conservation area ("Área de Conservación Privada") covering 589 hectares. It is on the south bank of the Rio Las Piedras and dominated by terra firme forest, which lacks the steep valleys associated with terra firme at other sites. Instead, it lies on top of a single flat terrace that tops out at 265 m above sea level. This region provides excellent habitat for the Brazil nut tree, *Bertholletia excelsa* and much of the deeper areas of LPBS are concessioned for Brazil nut harvest (Peres and Baider 1997, Cossio-Solano et al. 2011, Champagne 2024, Payne et al. 2024). Surprisingly, these terra firme regions are also dotted with small swamps, most of which are structured by the aguaje palm, *Mauritia flexuosa* L.F., (1782). Apart from the permanent swamps and mature terra firme forest, there are smaller areas of floodplain forest. The Amazon Research and Conservation Center (ARCC) (ca. 230 m a.s.l., on the north bank of the Rio Las Piedras) is situated on Lago Soledad, an oxbow lake of fascinating geography, with a near-complete 360-degree meander and a year-round connection to the main river through a single channel that drains both ends of the oxbow. The oxbow has rendered much of the region a seasonally flooded forest and predominantly a floodplain until hitting the high terraces of terra firma 5 km inland. This forest is more typically marked by the steep slopes of younger, less sandy terra firme and gives way to the valley of the Rio Huascar, 8 km to the north. Therefore, all study sites at ARCC lie between two major riverways and within 8 km of their confluence.

The wet season generally spans from October to April, characterised by almost daily rainfall and high humidity levels (90%) within forested areas. In contrast, the dry season, typically between May and October, brings warmer temperatures, with highs reaching up to 35°C, while humidity tends to be lower. Annual rainfall and temperature data (www.weather-atlas.com/en/peru/puerto-maldonado-climate) from the airport weather station in Puerto Maldonado indicate that the average annual temperature and precipitation are 29–32°C and 56.8–342.6 mm, respectively. Between May and August, the region experiences cold weather events known as "friajes." These events occur when cold winds move in from the south, causing temperatures to plummet to a range of +8 - +15 °C, lasting for up to 8 days.

Positional data was acquired in the field using GarminMap 64s (Model: 010-01199-10; Garmin Headquarters, Olathe, 1200 E 151st St, United States). Geographic coordinates within our dataset vary in spatial resolution dependent on the detection method. For pitfall traps, positions were acquired from the centre of the trap and represent the centroid of a 30 x 30 m cell. For transects, the radius is 71 m, for quadrats, the radius is 21 m. Due to the variable resolution and accuracy of opportunistic data, we extracted a representative position for the locality. We urge caution when examining opportunistic records, as they should be considered on a site-wide locality scale and will fall into one of two positions depending on the site where it was recorded. A small number of opportunistic records (n = 7) lack time and day-level date data.

### Coordinates

-12.1241 and -12.0020 Latitude; -69.6981 and -69.5139 Longitude.

## Taxonomic coverage

### Description

**Figure 2.**
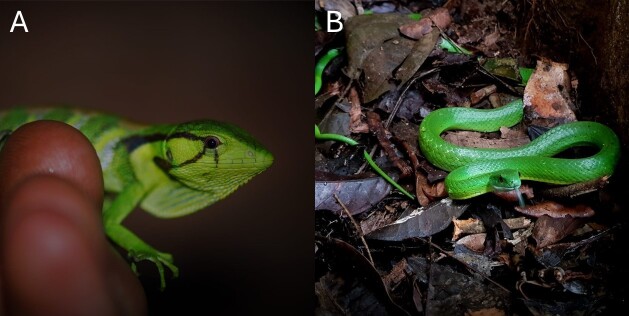
Some species, such as *Polychrus liogaster* **(A)** and *Chlorosoma viridissimum* **(B)**, were found opportunistically outside the principal study sites, although, within the overall locality. Photographs by Harry Turner.

Our dataset includes records of reptiles and amphibians from two localities near the Huascar and Las Piedras River confluences. Our dataset contains 2,327 distinct geo-referenced records, categorised into Anura (1,788), Crocodilia (10), Gymnophiona (1), Squamata (517) and Testudines (11). These records span 165 identified species, along with one entry recorded at the genus level (*Chironius*) (Table 2, Table 3). Six of the opportunistic records of five species were included from just outside the boundaries of the two study sites, but from within the overall study area locality and were included on our species list to provide a more complete coverage of documented species from the area (Fig. 2). An adult *Dracaena guianensis* (Daudin, 1802) was observed at a dried oxbow lake upriver from the two sites (coordinates -12.0608, -69.9127) in January, 2019. *Oscaecilia bassleri* (Dunn, 1942) , *Dendropsophus acreanus* (Bokermann, 1964) and *Chlorosoma viridissimum* (Linnaeus, 1758) were all found opportunistically to the south, but near the Las Piedras Biodiversity Station.

The amphibian inventory (Table 2), including prior reports, documented 79 species (ARCC = 64, LPBS = 69) representing 29 genera within 12 families across two orders. Anura dominated the assemblage with 78 species distributed amongst 28 genera and 11 families, while Gymnophiona was represented by a single caecilian species (*Oscaecilia bassleri*) in the family Caeciliidae. Amongst anurans, Hylidae was by far the most species-rich family with 41 species, followed by Leptodactylidae (11 species), Craugastoridae (9 species),and Microhylidae (5 species). Within Hylidae, the genus *Dendropsophus* showed the highest diversity with 15 species, while other notable genera included *Pristimantis* (7 species in Craugastoridae), *Leptodactylus* (8 species) and *Boana* (7 species). The remaining families each contributed fewer than five species: Aromobatidae (3 species), Bufonidae (3 species), Dendrobatidae (2 species), Centrolenidae (1 species), Ceratophryidae (1 species), Leiuperidae (1 species) and Pipidae (1 species). This taxonomic composition reflects a typical Neotropical lowland amphibian community dominated by arboreal frogs and direct-developing species. Notably, 18 species (23%) represent new records for the study area, indicating significant additions to the known amphibian fauna of this tributary system. New records include: *Rhaebo guttatus*, *Oreobates quixensis*, *Pristimantis imitatrix*, *P. ventrimarmoratus*, *Dendropsophus acreanus*, *D. bokermanni*, *D. joannae*, *D. leali*, *D. pauiniensis*, *D. salli*, *D. schubarti*, *Osteocephalus helenae*, *O. planiceps*, *Trachycephalus coriaceus*, *Leptodactylus bolivianus*, *Ctenophryne geayi*, *Pipa Pipa* and *Oscaecilia bassleri*.

The reptile inventory (Table 3), including prior reports, documented 96 species representing 66 genera within 24 families across three orders. Squamata was the most diverse order with 86 species distributed amongst 57 genera and 19 families, followed by Testudines with six species in six genera and four families and Crocodilia with four species in three genera within a single family (Alligatoridae). Among Squamata, snakes comprised the majority of diversity, with Dipsadidae being the most species-rich family (30 species in 20 genera), followed by Colubridae (10 species in 8 genera), Elapidae (5 species in 1 genus) and Viperidae (3 species in 2 genera). Lizard diversity was represented by multiple families, with notable contributions from Dactyloidae (5 Anolis species), Gymnophthalmidae (5 species in 3 genera), Tropiduridae (6 species in 3 genera) and Teiidae (5 species in 4 genera). The crocodilian assemblage included all three genera expected for the region: *Caiman* (1 species), *Melanosuchus* (1 species) and *Paleosuchus* (2 species). Turtle diversity encompassed four families typical of Amazonian freshwater habitats, with one species each of the genera *Mesoclemmys*, *Phrynops*, *Platemys*, *Kinosternon*, *Podocnemis* and *Chelonoidis*. Notably, 64 species (67%) represent new records for the study area, indicating substantial additions to the known reptile fauna of this tributary system. New records include: *Paleosuchus palpebrosus*, *Alopoglossus avilapiresae*, *Amphisbaena alba*, *Anolis punctatus*, *A. tandai*, *Bachia peruana*, *Cercosaura argulus*, *C. ocellata*, *Potamites ecpleopus*, *Enyalioides palpebralis*, *Polychrus liogaster*, *Varzea altamazonica*, *Gonatodes hasemanni*, *Pseudogonatodes guianensis*, *Dracaena guianensis*, *Kentropyx pelviceps*, *Plica umbra*, *Stenocercus prionotus*, *S. roseiventris*, *Urocentron azureum*, *Anilius scytale*, *Boa constrictor*, *Corallus batesii*, *Epicrates cenchria*, *Chironius exoletus*, *C. fuscus*, *C. scurrula*, *Chlorosoma viridissimum*, *Dendrophidion dendrophis*, *Oxybelis fulgidus*, *Rhinobothryum lentiginosum*, *Tantilla melanocephala*, *Adelphostigma occipitalis*, *Apostolepis nigroterminata*, *Atractus emmeli*, *A. major*, *A. snethlageae*, *Dipsas indica*, *Drymobius rhombifer*, *Drymoluber dichrous*, *Erythrolamprus aesculapii*, *E. taeniogaster*, *Helicops polylepis*, *Imantodes lentiferus*, *Leptophis ahaetulla*, *Oxyrhopus formosus*, *O. petolarius*, *Phrynonax poecilonotus*, *Pseudoboa coronata*, *Siphlophis cervinus*, *Xenodon severus*, *Xenoxybelis boulengeri*, *Micrurus annelatus*, *M. hemprichii*, *M. lemniscatus*, *M. obscurus*, *M. surinamensis*, *Amerotyphlops reticulatus*, *Bothrops atrox*, *B. bilineatus*, *Mesoclemmys gibba*, *Phrynops geoffroanus*, *Platemys platycephala* and *Kinosternon scorpioides*.

**Figure 3.**
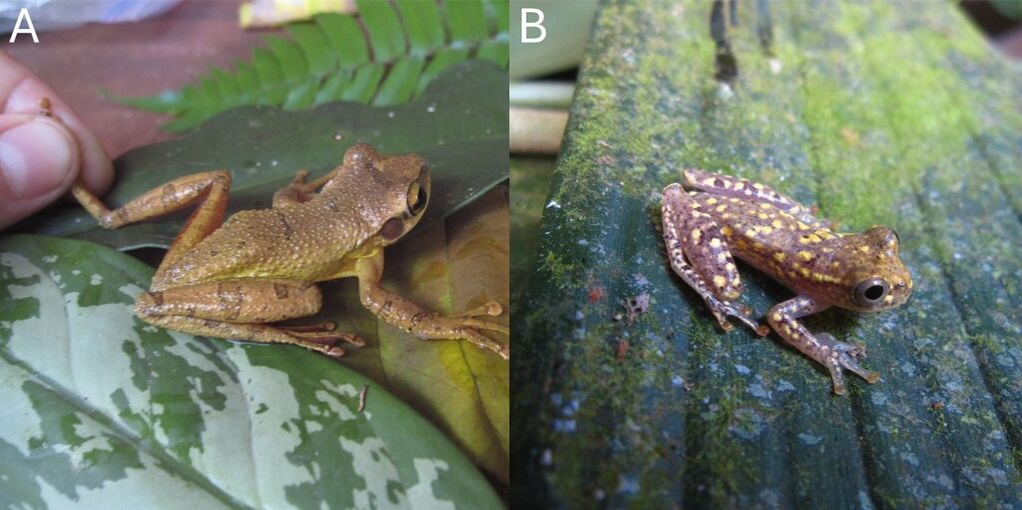
Examples of amphibian specimens from the Las Piedras Region illustrating taxonomic uncertainty and morphological variation. **A** Osteocephalus sp. from ARCC, displaying unmarked upper iris, yellow venter and tuberculate dorsal skin; **B** Dendropsophus gr. minutus from a swamp near LPBS, showing a densely spotted morph whose species identity remains uncertain due to polymorphism. Photographs by Brian Crnobrna.

Some records were removed because the identifications were inconsistent with known taxonomy or we were unable to identify them. The genus *Osteocephalus* presents several such specimens. *O. yasuni* was described in 1999 in distant Amazonian Ecuador (Ron and Pramuk 1999). Since then, the species has taken on variations observed in Peru (Moravec et al. 2016, Crnobrna et al. 2023). We removed records of *O. yasuni* (e.g. Fig. 3) we observed in Las Piedras, given the adverse biogeographic implications.

The genus *Chiasmocleis* (Méhelÿ, 1904) contains marked variations that likely occupy multiple species, which increases the potential of overlooked records of *C. shudikarensis* (Dunn, 1949) and/or *C. avilapiresae* (Peloso & Sturaro, 2008) from MDD. *Chiasmocleis supercilialba* (Morales & McDiarmid, 2009) was separated from *C. bassleri* (Dunn, 1949) by Morales and McDiarmid (2009). This contention has not been backed up by DNA evidence, but it is worth noting that one specimen from LPBS (a pitfall capture) fits the description of *C. supercilialba*, even though the population of *C. bassleri* was already established there by three consecutive research phases. Were it to be a separate species, its population would have been hidden from all survey efforts taking place less than 300 m away for two years of research. In any case, *Chiasmocleis* of this group are marked by their lack at most sites in MDD, which could shift IUCN classifications.

Integration of *Dendropsophus joannae* (Köhler & Lötters, 2001) into surveys took many years (Melo-Sampaio and de Souza 2015, Gagliardi Urrutia et al. 2022). The species appears to be widespread throughout Peruvian Amazonia. The *D. minutus* (Peters, 1872) species group contains cryptic species (Gehara et al. 2014, Orrico et al. 2021). One clue that may help to solidify candidate species is the presence of polymorphism expressed as a spotted morph (Fig. 3B) in D. gr. minutus, likely related to *D. delarivai* (Köhler & Lötters, 2001).

According to Fouquet et al. (2024), our records of *Rhinella* likely fall within *R. roqueana* of the *R. margaritifera* complex. However, as we are unable to verify this through images, we have assigned the Las Piedras specimens as *R. gr. margaritifera*. Additionally, according to Caminer et al. (2017), the *Dendropsophus* species occurring in Madre de Dios does not correspond to either *D. reticulatus* or *D. leucophyllatus*, but rather represents an undescribed taxon. Therefore, we refer to the population from Las Piedras as D. cf. leucophyllatus. Furthermore, the taxonomy of *Adenomera* is highly complex, with many populations once identified as *A. andreae* subsequently reclassified and described as distinct species (e.g. Zaracho et al. (2023)). Our photographs for *Adenomera* correspond to *A. hylaedactyla* based on snout length and, upon reviewing the collection, we revised the entry for as Piedras to *A. aff. hylaedactyla*. However, in the absence of call recordings, we cannot provide strong evidence for the occurrence of the remaining species.

Snakes of the genus *Chironius* (Fitzinger, 1826) were assigned to *C. exoletus* (Linnaeus, 1758) when keeled paravertebral scales were present. This is likely missing documented genetic variations. Torres-Carvajal et al. (2019) clade 3 remains unnamed. Future integrative studies must define: a) how keeled vertebral scales help in the diagnosis of the cryptic species and b) if records of *C. carinatus* from Brazilian sites similarly bear characteristics indicative of a cryptic identity/synonymy.

ARCC is a source for specimens of a poorly-defined species of blind snakes, which may be *Amerotyphlops brongersmianus* (Vanzolini, 1976). This snake differs from the more common *A. reticulatus* (Linnaeus, 1758) by having a uniform brown pattern and an imbricate tail calcar. The occurrence of this snake was restricted to the first half-kilometre of Trail A at ARCC. In this forest the trees — mostly palms — do not grow very tall, exposing soil to increased solar heating and the soil is notably sandy, i.e. a good substrate for reptile eggs. This is due to riverine deposition from the Las Piedras, which contains Trail A in a small 100 × 500 m area bounded by river and lake. An Amerotyphlops cf. brongersmianus was collected from a shebon palm (*Attalea* sp.) growing in the region during a quadrat search. The snake had taken refuge below one of the tree’s shed leaf scales, and was extracted from approx. 1.3 m height off the ground. This counterintuitive preference of blindsnakes for aerial habitats has been noted in other genera (Vanzolini 1970, Gehlbach et al. 1971, Webb and Shine 1992, Das and Wallach 1998, Landestoy 2023), and may sufficiently differentiate A. cf. bongersmianus niche from *A. reticulatus*, which was also captured via pitfall trapping on Trail A. The lizard *Stenocercus prionotus* (Cadle, 2001) was also found during a quadrat search on Trail A. Comments by Cadle (2001) that the species occurs in secondary forest and farm fields belie the rarity of records and cannot be interpreted as a diminished conservation concern. This was the only record in all the data amassed from Las Piedras. Records from the Tambopata and Manu Basins are likewise scarce. When paired with absence at several intensely studied sites (Duellman 2005, Rabosky et al. 2019), the natural history of the lizard cannot easily be characterised and may be better served in the data-deficient category of the IUCN (Gärdenfors et al. 2001). The rather extreme reptile species accumulation on Trail A included coral snakes. Both the typical red-banded and the red-and-white-banded morphs of *Micrurus annelatus* (Peters, 1871) were seen and a sole observation of *M. hemprichii* (Jan, 1858) (a juvenile). *M. lemniscatus* (Linnaeus, 1758) is very common at the Las Piedras Biodiversity Station. In fact, some manner of rookery or communal nest site was located in a small patch of seasonally flooded forest near the station, involving 10 individuals.

### Taxa included

**Table:** 

Rank	Scientific Name	Common Name
kingdom	Animalia	Animals
subkingdom	Eumetazoa	
phylum	Chordata	
subphylum	Vertebrata	
class	Amphibia	Amphibians
class	Reptilia	Reptiles
superorder	Lepidosauria	
order	Crocodylia	Crocodilians
order	Testudines	Turtles
order	Squamata	Snakes and Lizards
order	Anura	Frogs
order	Gymnophiona	Caecilians
family	Aromobatidae	Cryptic forest frogs
genus	*Allobates* (Zimmermann & Zimmermann, 1988)	
species	*Allobates femoralis* (Boulenger, 1884)	Brilliant-thighed poison frog
species	*Allobates trilineatus* (Boulenger, 1884)	Three-striped rocket frog
species	*Allobates conspicuus* (Morales, 2002)	
family	Bufonidae	True toads
genus	*Rhaebo* (Cope, 1862)	
species	*Rhaebo guttatus* (Schneider, 1799)	Smooth-sided toad
genus	*Rhinella* (Fitzinger, 1826)	South American toads
species	*Rhinella margaritifera* (Schneider, 1799)	South American common toad
species	*Rhinella marina* (Linnaeus, 1758)	Cane toad
family	Centrolenidae	Glass frogs
genus	Hyalinobatrachium (Ruiz-Carranza & Lynch, 1991)	
species	*Hyalinobatrachium mondolfii* (Señaris & Ayarzagüena, 2001)	
family	Ceratophryidae	Common horned frogs
genus	*Ceratophrys* (Wied-Neuwied, 1824)	South American horned frogs
species	*Ceratophrys cornuta* (Linnaeus, 1758)	Surinam horned frog
family	Craugastoridae	Fleshbelly frogs
genus	*Oreobates* (Jiménez de la Espada, 1872)	
species	*Oreobates cruralis* (Boulenger, 1902)	La Paz robber frog
species	*Oreobates quixensis* (Jiménez de la Espada, 1872)	Common big-headed frog
genus	*Pristimantis* (Jiménez de la Espada, 1870)	
species	*Pristimantis altamazonicus* (Barbour & Dunn, 1921)	
species	*Pristimantis fenestratus* (Steindachner, 1864)	Rio Mamore robber frog
species	*Pristimantis imitatrix* (Duellman, 1978)	
species	*Pristimantis reichlei* (Padial & De la Riva, 2009)	
species	*Pristimantis peruvianus* (Flores & Rodríguez, 1997)	Peruvian rain frog
species	*Pristimantis toftae* (Duellman, 1978)	
species	*Pristimantis ventrimarmoratus* (Boulenger, 1912)	
family	Dendrobatidae	Poison dart frogs
genus	*Ameerega* (Bauer, 1986)	
species	*Ameerega hahneli* (Boulenger, 1884)	
species	*Ameerega trivittata* (Spix, 1824)	Three-striped poison frog
family	Hylidae	Tree frogs
genus	*Boana* (Gray, 1825)	Gladiator frogs
species	*Boana boans* (Linnaeus, 1758)	Giant gladiator treefrog
species	*Boana cinerascens* (Spix, 1824)	Rough-skinned green treefrog
species	*Boana fasciata* (Günther, 1858)	Gunther's banded treefrog
species	*Boana geographica* (Spix, 1824)	Map tree frog
species	*Boana lanciformis* (Cope, 1871)	Basin tree frog
species	*Boana punctata* (Schneider, 1799)	Polka-dot treefrog
species	*Boana steinbachi* (Boulenger, 1905)	Sara tree frog
genus	*Cruziohyla* (Faivovich et al., 2005)	
species	*Cruziohyla craspedopus* (Funkhouser, 1957)	Fringed leaf frog
genus	*Dendropsophus* (Fitzinger, 1843)	Fitzinger neotropical treefrogs
species	*Dendropsophus acreanus* (Bokermann, 1964)	Acre treefrog
species	*Dendropsophus bokermanni* (Goin, 1960)	Bokermann's Tarauaca treefrog
species	*Dendropsophus brevifrons* (Duellman & Crump, 1974)	Dendropsophus brevifrons
species	*Dendropsophus joannae*	Dendropsophus joannae
species	*Dendropsophus pauiniensis* (Heyer, 1977)	
species	*Dendropsophus leali* (Bokermann, 1964)	Plain-colored treefrog
species	*Dendropsophus reticulatus* (Jiménez de la Espada, 1870)	The reticulated treefrog
species	*Dendropsophus kamagarini* (Rivadeneira, Venegas & Ron, 2018)	
species	*Dendropsophus rhodopeplus* (Günther, 1858)	Red-skirted treefrog
species	*Dendropsophus salli* (Jungfer, Reichle & Piskurek, 2010)	
species	*Dendropsophus sarayacuensis* (Shreve, 1935)	Shreve's Sarayacu treefrog
species	*Dendropsophus schubarti* (Bokermann, 1963)	Schubart's Rondonia treefrog
species	*Dendropsophus timbeba* (Martins & Cardoso, 1987)	Cardoso's treefrog
species	*Dendropsophus triangulum* (Günther, 1869)	Variable clown treefrog
species	*Dendropsophus minutus* (Peters, 1872)	Lesser treefrog
genus	*Osteocephalus* (Steindachner, 1862)	Slender-legged treefrogs
species	*Osteocephalus castaneicola* (Moravec, Aparicio, Guerrero-Reinhard, Calderón, Jungfer & Gvozdík, 2009)	Bolivian spiny-backed frog
species	*Osteocephalus helenae* (Ruthven, 1919)	Urubamba spiny-backed frog
species	*Osteocephalus planiceps* (Cope, 1874)	Flat-headed spiny-backed frog
species	*Osteocephalus taurinus* (Steindachner, 1862)	Manaus slender-legged treefrog
genus	*Callimedusa* (Duellman, Marion & Hedges, 2016)	
species	*Callimedusa atelopoides* (Duellman, Cadle & Cannatella, 1988)	Toady leaf frog
species	*Callimedusa tomopterna* (Cope, 1868)	Tiger-striped treefrog
genus	*Phyllomedusa* (Wagler, 1830)	Monkey frogs
species	*Phyllomedusa bicolor* (Boddaert, 1772)	Giant monkey frog
species	*Phyllomedusa camba* (De la Riva, 1999)	Black-eyed monkey frog
species	*Phyllomedusa palliata* (Peters, 1873)	Waxy monkey frog
species	*Phyllomedusa vaillantii* (Boulenger, 1882)	White-lined leaf frog
genus	*Scarthyla* (Duellman & de Sá, 1988)	
species	*Scarthyla goinorum* (Bokermann, 1962)	Tarauaca snouted treefrog
genus	*Scinax* (Wagler, 1830)	Snouted treefrogs
species	*Scinax garbei* (Miranda-Ribeiro, 1926)	Eirunepe snouted treefrog
species	*Scinax ictericus* (Duellman & Wiens, 1993)	
species	*Scinax pedromedinae* (Henle, 1991)	Henle's snouted treefrog
species	*Scinax ruber* (Laurenti, 1768)	Red snouted treefrog
genus	*Sphaenorhynchus* (Tschudi, 1838)	Hatchet-faced treefrogs
species	*Sphaenorhynchus lacteus* (Daudin, 1800)	Greater hatchet-faced treefrog
genus	*Trachycephalus* (Tschudi, 1838)	Casque-headed tree frogs
species	*Trachycephalus coriaceus* (Peters, 1867)	Surinam golden-eyed treefrog
species	*Trachycephalus typhonius* (Linnaeus, 1758)	Veined treefrog
subfamily	Leiuperinae	
genus	*Edalorhina* (Jiménez de la Espada, 1870)	Snouted frogs
species	*Edalorhina perezi* (Jiménez de la Espada, 1870)	Perez's snouted frog
family	Leptodactylidae	Foam-nest frogs
genus	*Adenomera* (Steindachner, 1867)	Tropical bullfrogs
species	*Adenomera andreae* (Müller, 1923)	Lowland tropical bullfrog
genus	*Engystomops* (Jiménez de la Espada, 1872)	Túngara frogs
species	*Engystomops petersi* (Steindachner, 1864)	Peter's dwarf frog
genus	*Leptodactylus* (Fitzinger, 1826)	
species	*Leptodactylus bolivianus* (Boulenger, 1898)	Bolivian white-lipped frog
species	*Leptodactylus didymus* (Heyer, García-Lopez & Cardoso, 1996)	Madre de Dios thin-toed frog
species	*Leptodactylus knudseni* (Heyer, 1972)	Knudsen's frog
species	*Leptodactylus leptodactyloides* (Andersson, 1945)	
species	*Leptodactylus pentadactylus* (Laurenti, 1768)	Smoky jungle frog
species	*Leptodactylus petersii* (Steindachner, 1864)	Peters' thin-toed frog
species	*Leptodactylus rhodomystax* (Boulenger, 1884)	Loreto white-lipped frog
species	*Leptodactylus rhodonotus* (Günther, 1869)	Peru White-lipped Frog
genus	*Lithodytes* (Fitzinger, 1843)	Antnest frog
species	*Lithodytes lineatus* (Schneider, 1799)	Painted antnest frog
family	Microhylidae	Narrow-mouthed frogs
genus	*Chiasmocleis* (Méhelÿ, 1904)	Humming frogs
species	*Chiasmocleis bassleri* (Dunn, 1942)	Bassler's humming frog
species	*Chiasmocleis ventrimaculata* (Andersson, 1945)	Dotted humming frog
genus	*Ctenophryne* (Mocquard, 1904)	Egg frogs
species	*Ctenophryne geayi* (Mocquard, 1904)	Brown egg frog
genus	*Elachistocleis* (Parker, 1927)	Oval frogs
species	*Elachistocleis muiraquitan* (Nunes-de-Almeida & Toledo, 2012)	Acre's oval frog
genus	*Hamptophryne* (Carvalho, 1954)	Bleating frogs
species	*Hamptophryne boliviana* (Parker, 1927)	Bolivian bleating frog
family	Pipidae	Clawed frogs
genus	*Pipa* (Laurenti, 1768)	Surinam toads
species	*Pipa pipa* (Linnaeus, 1758)	Surinam toad
family	Caeciliidae	Common caecilians
genus	*Oscaecilia* (Taylor, 1968)	South American caecilians
species	*Oscaecilia bassleri* (Dunn, 1942)	Pastaza River caecilian
family	Alligatoridae	Alligators and Caiman
genus	*Caiman* (Spix, 1825)	
species	*Caiman crocodilus* (Linnaeus, 1758)	Spectacled caiman
genus	*Melanosuchus* (Gray, 1862)	
species	*Melanosuchus niger* (Spix, 1825)	Black caiman
genus	*Paleosuchus* (Gray, 1862)	
species	*Paleosuchus palpebrosus* (Cuvier, 1807)	Cuvier's dwarf caiman
species	*Paleosuchus trigonatus* (Schneider, 1801)	Schneider's smooth-fronted caiman
family	Alopoglossidae	
genus	*Alopoglossus* (Boulenger, 1885)	
species	*Alopoglossus avilapiresae* (Ibeiro-Júnior, Choueri, Lobos, Venegas, Torres-Carvajal & Werneck, 2020)	
family	Amphisbaenidae	Worm lizards
genus	*Amphisbaena* (Linnaeus, 1758)	Worm lizards
species	*Amphisbaena alba* (Linnaeus, 1758)	White-bellied worm lizard
family	Dactyloidae	Anoles
genus	*Anolis* (Daudin, 1802)	Anoles
species	*Anolis fuscoauratus* (d'Orbigny, 1837)	Slender anole
species	*Anolis punctatus* (Daudin, 1802)	Amazon green anole
species	*Anolis tandai* (Avila-Pires, 1995)	Tanda's anole
family	Polychrotidae	Bush anoles
genus	*Polychrus* (Cuvier, 1817)	Bush anoles
species	*Polychrus liogaster* (Boulenger, 1908)	Boulenger's bush anole
family	Diploglossidae	Galliwasps
genus	*Diploglossus* (Wiegmann, 1834)	Galliwasps
species	*Diploglossus fasciatus* (Gray, 1831)	Banded Galliwasp
family	Gymnophthalmidae	Spectacled lizards
genus	*Bachia* (Gray, 1845)	Bachia
species	*Bachia peruana* (Werner, 1901)	Peru bachia
genus	*Cercosaura* (Wagler, 1830)	
species	*Cercosaura argulus* (Peters, 1862)	The elegant eyed-lizard
species	*Cercosaura eigenmanni* (Griffin, 1917)	Eigenmann's prionodactylus
species	*Cercosaura ocellata* (Wagler, 1830)	Ocellated tegu
genus	*Potamites* (Doan & Castoe, 2005)	Stream-lizards
species	*Potamites ecpleopus* (Cope, 1875)	Common stream-lizard
family	Hoplocercidae	Dwarf iguanas
genus	*Enyalioides* (Boulenger, 1885)	Woodlizards
species	*Enyalioides palpebralis* (Boulenger, 1883)	Horned woodlizard
family	Phyllodactylidae	Leaf-toed geckos
genus	*Thecadactylus* (Goldfuss, 1820)	Turnip-tailed geckos
species	*Thecadactylus solimoensis* (Houttuyn, 1782)	Southern turniptail gecko
family	Scincidae	Skinks
genus	*Varzea* (Hedges & Conn, 2012)	
species	*Varzea altamazonica* (Miralles, Barrio-Amoros, Rivas & Chaparro-Auza, 2006)	Upper-Amazon Skink
family	Sphaerodactylidae	Least Geckos
genus	*Gonatodes* (Fitzinger, 1843)	
species	*Gonatodes hasemanni* (Griffin, 1917)	Haseman's gecko
species	*Gonatodes humeralis* (Guichenot, 1855)	Trinidad gecko
genus	*Pseudogonatodes* (Ruthven, 1915)	South American clawed geckos
species	*Pseudogonatodes guianensis* (Parker, 1935)	Amazon Pygmy Gecko
family	Teiidae	Tegus
genus	*Ameiva* (Meyer, 1795)	Jungle-runners
species	*Ameiva ameiva* (Linnaeus, 1758)	Amazon racerunner
genus	*Kentropyx* (Spix, 1825)	Whiptails
species	*Kentropyx altamazonicus* (Cope, 1875)	Cocha whiptail
species	*Kentropyx pelviceps* (Cope, 1868)	Forest whiptail
genus	*Tupinambis* (Daudin, 1802)	Tegus
species	*Tupinambis cuzcoensis* (Murphy, Jowers, Lehtinen, Charles, Colli, Peres Jr, Hendry & Pyron, 2016)	Cusco tegu
genus	*Dracaena* (Daudin 1802)	Caiman lizards
species	*Dracaena guianensis* (Daudin, 1801)	Northern caiman lizard
family	Tropiduridae	Neotropical ground lizards
genus	*Plica* (Gray, 1831)	Tree-runner
species	*Plica plica* (Linnaeus, 1758)	Collared tree-runner
species	*Plica umbra* (Linnaeus, 1758)	Blue-lipped tree lizard
genus	*Stenocercus* (Duméril & Bibron, 1837)	Whorltail iguanas
species	*Stenocercus fimbriatus* (Avila-Pires, 1995)	Western leaf lizard
species	*Stenocercus prionotus* (Cadle, 2001)	
species	*Stenocercus roseiventris* (d'Orbigny in Duméril & Bibron, 1837)	Rose whorltail iguana
genus	*Urocentron* (Kaup, 1827)	Thornytail iguana
species	*Urocentron azureum* (Linnaeus, 1758)	Green thornytail iguana
family	Aniliidae	Pipe snake
genus	*Anilius* (Oken, 1816)	American pipe snake
species	*Anilius scytale* (Linnaeus, 1758)	American pipe snake
family	Boidae	Boas
genus	*Boa* (Linnaeus, 1758)	Boas
species	*Boa constrictor* (Linnaeus, 1758)	Boa constrictor
genus	*Corallus* (Daudin, 1803)	Neotropical tree boas
species	*Corallus hortulanus* (Linnaeus, 1758)	Amazon tree boa
species	*Corallus batesii* (Gray, 1860)	Bates’ emerald tree-boa
genus	*Epicrates* (Wagler, 1830)	Rainbow boas
species	*Epicrates cenchria* (Linnaeus, 1758)	Rainbow boa
genus	*Eunectes* (Wagler, 1830)	Anacondas
species	*Eunectes murinus* (Linnaeus, 1758)	Green Anaconda
family	Colubridae	Colubrids
genus	*Chironius* (Fitzinger, 1826)	Sipos
species	*Chironius exoletus* (Linnaeus, 1758)	Linnaeus's sipo
species	*Chironius fuscus* (Linnaeus, 1758)	Brown Whipsnake
species	*Chironius scurrula* (Wagler, 1824)	The Rusty Whipsnake
genus	*Chlorosoma* (Wagler, 1830)	
species	*Chlorosoma viridissimum* (Linnaeus, 1758)	Common green racer
genus	*Dendrophidion* (Fitzinger, 1843)	Forest racers
species	*Dendrophidion dendrophis* (Schlegel, 1837)	Olive Forest-Racer
genus	*Drymarchon* (Fitzinger, 1843)	Indigo snakes
species	*Drymarchon corais* (Boie, 1827)	Yellow-tail cribo
genus	*Oxybelis* (Wagler, 1830)	Vine snakes
species	*Oxybelis fulgidus* (Daudin, 1803)	Green vine snake
genus	*Rhinobothryum* (Wagler, 1830)	
species	*Rhinobothryum lentiginosum* (Scopoli, 1785)	Amazon banded snake
genus	*Spilotes* (Wagler, 1830)	
species	*Spilotes pullatus* (Linnaeus, 1758)	Chicken snake
genus	*Tantilla* (Baird & Girard, 1853)	Centipiede snakes
species	*Tantilla melanocephala* (Cope, 1861)	Black-headed centipede snake
genus	*Xenoxybelis* (Machado, 1993)	Sharpnose snake
species	*Xenoxybelis boulengeri* (Procter, 1923)	Southern sharpnose snake
genus	*Apostolepis* (Cope, 1862)	Burrowing snakes
species	*Apostolepis nigroterminata* (Boulenger, 1896)	Peru burrowing snake
genus	*Atractus* (Wagler, 1828)	Ground snakes
species	*Atractus major* (Boulenger, 1894)	Greater Ground Snake
species	*Atractus snethlageae* (Cunha & Nascimento, 1983)	
species	*Atractus emmeli* (Boettger, 1888)	Emmel's ground snake
genus	*Clelia* (Fitzinger, 1826)	Mussurana
species	*Clelia clelia* (Daudin, 1803)	Black mussurana
genus	*Dipsas* (Laurenti, 1768)	Snail-eaters
species	*Dipsas catesbyi* (Sentzen, 1796)	Catesby's snail-eater
species	*Dipsas indica* (Laurenti, 1768)	Neotropical snail-eater
genus	*Drepanoides* (Dunn, 1928)	Black-collared snake
species	*Drepanoides anomalus* (Jan, 1863)	Black-collared snake
genus	*Drymobius* (Fitzinger, 1843)	Neotropical racers
species	*Drymobius rhombifer* (Günther, 1860)	Esmarald racer
genus	*Drymoluber* (Amaral, 1930)	Woodland racers
species	*Drymoluber dichrous* (Günther, 1860)	Northern woodland racer
genus	*Erythrolamprus* (Boie, 1826)	False coral snakes
species	*Erythrolamprus aesculapii* (Linnaeus, 1758)	Aesculapian false coral snake
species	*Erythrolamprus reginae* (Linnaeus, 1758)	Royal Ground Snake
species	*Erythrolamprus taeniogaster* (Jan, 1863)	
kingdom	Imantodes (Duméril, 1853)	Blunt-headed vine snakes
species	*Imantodes cenchoa* (Linnaeus, 1758)	Blunt-headed vine snake
species	*Imantodes lentiferus* (Cope, 1894)	Amazon Basin tree snake
genus	*Helicops* (Wagler, 1828)	South-American keelbacks
species	*Helicops angulatus* (Linnaeus, 1758)	Brown-banded watersnake
species	*Helicops polylepis* (Günther, 1861)	Norman's keelback
genus	*Leptodeira* (Fitzinger, 1843)	Cat-eyed snakes
species	*Leptodeira annulata* (Linnaeus, 1758)	Banded cat-eyed snake
genus	*Leptophis* (Bell, 1825)	Parrot snakes
species	*Leptophis ahaetulla* (Linnaeus, 1758)	Parrot snake
genus	*Oxyrhopus* (Wagler, 1830)	False coral snakes
species	*Oxyrhopus formosus* (Wied-neuwied, 1820)	Formosa false coral snake
species	*Oxyrhopus melanogenys* (Tschudi, 1845)	Tschudi's false coral snake
species	*Oxyrhopus petolarius* (Linnaeus, 1758)	Forest flame snake
genus	*Xenoxybelis* (Machado, 1993)	Sharpnose snakes
species	*Xenoxybelis boulengeri* (Procter, 1923)	Southern sharpnose snake
genus	*Phrynonax* (Cope, 1862)	
species	*Phrynonax poecilonotus* (Günther, 1858)	Puffing snake
genus	*Pseudoboa* (Schneider, 1801)	False boa
species	*Pseudoboa coronata* (Schneider, 1801)	Crowned false boa
genus	*Siphlophis* (Fitzinger, 1843)	Night snakes
species	*Siphlophis compressus* (Daudin, 1803)	Tropical flat snake
species	*Siphlophis cervinus* (Laurenti, 1768)	Panamanian spotted night
genus	*Adelphostigma* (Abegg, Santos-Jr, Costa, Battilana, Graboski, Vianna, Azevedo, Fagundes, Castille, Prado, Bonatto, Zaher & Grazziotin, 2008)	
species	*Adelphostigma occipitalis* (Jan, 1863)	
genus	*Xenodon* (Boie, 1826)	False fer-de-lances
species	*Xenodon severus* (Linnaeus, 1758)	Amazon false fer-de-lance
genus	*Xenopholis* (Peters, 1869)	Ground snakes
species	*Xenopholis scalaris* (Wucherer, 1861)	Wucherer's ground snake
family	Elapidae	Elapids
genus	*Micrurus* (Wagler, 1824)	Coral snakes
species	*Micrurus annelatus* (Peters, 1871)	Annellated coral snake
species	*Micrurus hemprichii* (Jan, 1858)	Hemprichi's coral snake
species	*Micrurus lemniscatus* (Linnaeus, 1758)	South American coral snake
species	*Micrurus obscurus* (Jan, 1872)	Black-neck Amazonian coral snake
species	*Micrurus surinamensis* (Cuvier, 1816)	Aquatic coral snake
family	Typhlopidae	Blind snakes
genus	*Amerotyphlops* (Hedges, Marion, Lipp, Marin & Vidal, 2014)	
species	*Amerotyphlops reticulatus* (Linnaeus, 1758)	Reticulated worm snakes
family	Viperidae	Vipers
genus	*Bothrops* (Wagler, 1824)	Lanceheads
species	*Bothrops atrox* (Linnaeus, 1758)	Common lancehead
species	*Bothrops bilineatus* (Wied-Neuwied, 1821)	Palm viper
genus	*Lachesis* (Daudin, 1803)	Bushmasters
species	*Lachesis muta* (Linnaeus, 1766)	South American bushmaster
family	Chelidae	Austro-South American side-neck turtles
genus	*Platemys* (Wagler, 1830)	
species	*Platemys platycephala* (Schneider, 1792)	Flat-headed turtle
genus	*Mesoclemmys* (Gray, 1873)	Toadhead turtles
species	*Mesoclemmys gibba* (Schweigger, 1812)	Toadhead turtle
genus	*Phrynops* (Wagler, 1830)	Bearded toadheads
species	*Phrynops geoffroanus* (Schweigger, 1812)	Geoffroy's side-necked turtle
genus	*Kinosternon* (Spix, 1824)	Mud Turtles
species	*Kinosternon scorpioides* (Linnaeus, 1766)	Scorpion mud turtle
genus	*Podocnemis* (Wagler, 1830)	South-American mud turtles
species	*Podocnemis unifilis* (Troschel, 1848)	Yellow-spotted river turtle
genus	*Chelonoidis* (Fitzinger, 1835)	Tortoise
species	*Chelonoidis denticulatus* (Linnaeus, 1766)	Yellow-footed tortoise

## Temporal coverage

**Data range:** 2004-5-01 – 2024-10-01; 2012-5-01 – 2013-12-20.

### Notes

Opportunistic records were acquired between 2004 to 2024. Pitfall, transect and quadrat data were acquired between 2012 and 2013.

## Usage licence

### Usage licence

Other

### IP rights notes

CC-BY 4.0

## Data resources

### Data package title

Occurrence dataset of reptiles and amphibians in primary forests of the Las Piedras River, Tambopata Province, Peru

### Resource link

https://doi.org/10.15468/sa8m3q

### Alternative identifiers

https://ipt.pensoft.net/resource?r=herpetofauna_las_piedras_peru

### Number of data sets

1

### Data set 1.

#### Data set name

Occurrence dataset of reptiles and amphibians in primary forests of the Las Piedras River, Tambopata Province, Peru

#### Download URL

https://ipt.pensoft.net/archive.do?r=herpetofauna_las_piedras_peru

#### Description

This dataset contains 2327 occurrence records of 165 species belonging to 33 families and 5 orders.

- **Order: Anura (Frogs and Toads)**:
  - Families: Aromobatidae (2 species), Bufonidae (3 species), Ceratophrynidae (1 species), Craugastoridae (9 species), Dendrobatidae (2 species), Hylidae (40 species), Leiuperidae (1 species), Leptodactylidae (11 species), Microhylidae (5 species) and Pipidae (1 species).
- **Order: Crocodilia (Crocodilians)**:
  - Family: Alligatoridae (4 species).
- **Order: Gymnophiona (Caecilians)**:
  - Family: Caeciliidae (1 species).
- **Order: Squamata (Lizards, Snakes and Amphisbaenians)**:
  - Families: Dipsadidae (27 species), Colubridae (12 species), Hylidae (40 species), Boidae (5 species) and others.
- **Order: Testudines (Turtles)**:
  - Families: Chelidae (2 species), Kinosternidae (1 species), Testudinidae (1 species).

**Data set 1.** 

Column label	Column description
datasetName	The name of the dataset to which the record belongs (e.g. "Occurrence dataset of reptiles and amphibians in primary forests of the Las Piedras River, Tambopata Province, Peru").
occurrenceID	A unique identifier for each occurrence record.
language	The language used in the dataset (e.g. "en" for English).
basisOfRecord	The type of data collection method (e.g. "Occurrence").
recordedBy	The name(s) of the person(s) who recorded the observation.
recordedByID	ORCID or other identifiers for the observer(s), if available.
occurrenceStatus	The status of the observation (e.g. "present").
order	The taxonomic order of the species observed (e.g. Anura, Squamata).
family	The taxonomic family of the species observed (e.g. Hylidae, Dipsadidae).
scientificName	The scientific name of the species (e.g. *Adelphostigma occipitalis*).
scientificNameAuthorship	The authority who formally described the species.
dynamicProperties	Conservation status of the species as of 2024, according to the International Union for Conservation of Nature (e.g. "Least Concern").
eventDate	The date when the observation was made, in ISO format, is as follows: (YYYY-MM-DD).
eventTime	The time of day when the observation was recorded.
countryCode	The ISO country code for the observation location (e.g. "PE" for Peru).
taxonRank	The taxonomic rank of the observed entity (e.g. "species").
kingdom	The kingdom of the organism (e.g. "Animalia").
decimalLatitude	The latitude of the observation location in decimal degrees.
decimalLongitude	The longitude of the observation location in decimal degrees.
geodeticDatum	The geodetic datum used for the spatial coordinates (e.g. "WGS84").
individualCount	The number of individuals observed in the record.
country	The name of the country where the observation was made (e.g. "Peru").
samplingProtocol	The method or protocol used to collect the data (e.g. "opportunistic sampling").
locality	The specific locality of the observation (e.g. "Next to transect at the Las Piedras Biodiversity Station, Pitfall trap was located on upper ridge overlooking dropoff towards the Las Piedras River. Location is 160 m inland from the river").
coordinateUncertaintyInMetres	The uncertainty in the spatial coordinates, measured in metres.
dataGeneralisations	Modifications, simplifications or transformations applied to a dataset to make it more general, less detailed or more suitable for specific analytical purposes.
genus	This field contains the genus name of the organism (e.g. Adelphostigma).

## Figures and Tables

**Table 1.** Summary of sampling effort and methods used for herpetofaunal surveys at Las Piedras Biodiversity Station (LPBS) and Amazon Research and Conservation Centre (ARCC) in Madre de Dios, Peru. Diurnal sampling effort represents the percentage of total visual encounter surveys (transects and quadrat searches) conducted during daylight hours. Sampling methods included standardised transect surveys, quadrat searches of varying sizes, pitfall trap arrays with drift fences and opportunistic time-constrained searches. Trap/hours represent total sampling effort across all pitfall traps, while person/days indicate the cumulative field effort for opportunistic encounters.

Method	Site	Samples	% diurnal sampling	Effort
100 m transects				
	LPBS	153	44%	128 man/hours
	ARCC	64	13%	103 man/hours
Quadrat searches				
	LPBS	123 (88 100 m^2^ quadrats, 35 400 m^2^)	14%	169 man/hours
	ARCC	12 (400 m^2^)	58%	32 man/hours
Pitfall traps				
	LPBS	8 traps (220 m total driftline w/ 30 buckets)		5,491 trap/hours
	ARCC	8 traps (260 m total driftline w/ 34 buckets)		8,378 trap/hours
Time-constrained searches (opportunistic sampling)				
	LPBS	74 days		242 person/days
	ARCC	118 days		324 person/days

**Table 2.** Amphibian species found on the Las Piedras River, Madre de Dios, Peru. Most records were documented at Las Piedras Biodiversity Station (LPBS) and the Amazon Research and Conservation Centre (ARCC). * Denotes records not found in our dataset, but confirmed to occur on the Las Piedras Tributary by reviewing all vertebrate literature from the study area. International Union for Conservation of Nature (IUCN 20225-1) designations denoted as: LC = least concern, DD = data deficient, NE = Not evaluated, VU = vulnerable.

Order	Family	Genus	Specific epithet	LPBS	ARCC	Previously reported on the tributary by	New records	IUCN Designation
Anura	Aromobatidae	* Allobates *	*conspicuous*	X*		von May et al. (2009), Tatum-Hume et al. (2003) (reported as *Colostethus conspicuus*)		LC
Anura	Aromobatidae	* Allobates *	* femoralis *	X	X	von May et al. (2009), Tatum-Hume et al. (2003) (reported as *Epipedobates femoralis*)		LC
Anura	Aromobatidae	* Allobates *	* trilineatus *	X	X	von May et al. (2009)		LC
Anura	Bufonidae	* Rhaebo *	* guttatus *	X			X	LC
Anura	Bufonidae	* Rhinella *	gr. margaritifera	X	X	von May et al. (2009), Tatum-Hume et al. (2003) (reported as *Bufo typhonius*)		LC
Anura	Bufonidae	* Rhinella *	* marina *	X	X	von May et al. (2009), Tatum-Hume et al. (2003) (reported as *Bufo marinus*)		LC
Anura	Centrolenidae	* Hyalinobatrachium *	* mondolfii *	X*		Chavez et al. (2019)		LC
Anura	Ceratophryidae	* Ceratophrys *	* cornuta *	X	X	von May et al. (2009)		LC
Anura	Craugastoridae	* Oreobates *	* cruralis *	X	X	Tatum-Hume et al. (2003)(reported as Eleutherodactylus cf. cruralis)		LC
Anura	Craugastoridae	* Oreobates *	* quixensis *		X		X	LC
Anura	Craugastoridae	* Pristimantis *	* altamazonicus *	X	X	von May et al. (2009), Tatum-Hume et al. (2003) (reported as *Eleutherodactylus altamazonicus*)		LC
Anura	Craugastoridae	* Pristimantis *	* fenestratus *	X	X	von May et al. (2009), Tatum-Hume et al. (2003) (reported as *Eleutherodactylus fenestrataus*)		LC
Anura	Craugastoridae	* Pristimantis *	* imitatrix *		X		X	LC
Anura	Craugastoridae	* Pristimantis *	* peruvianus *	X		von May et al. (2009), Tatum-Hume et al. (2003) (reported as *Eleutherodactylus peruvianus*)		LC
Anura	Craugastoridae	* Pristimantis *	* reichlei *	X	X	von May et al. (2009)		NE
Anura	Craugastoridae	* Pristimantis *	* toftae *	X	X	von May et al. (2009), Tatum-Hume et al. (2003) (reported as *Eleutherodactylus toftae*)		LC
Anura	Craugastoridae	* Pristimantis *	* ventrimarmoratus *	X		von May et al. (2009)	X	LC
Anura	Dendrobatidae	* Ameerega *	* hahneli *	X	X	von May et al. (2009), Tatum-Hume et al. (2003) (reported as *Epipedobates hahneli*)		LC
Anura	Dendrobatidae	* Ameerega *	* trivittata *	X	X	von May et al. (2009), Tatum-Hume et al. (2003) (reported as *Epipedobates trivittatus*)		LC
Anura	Hylidae	* Boana *	* boans *	X	X	von May et al. (2009), Tatum-Hume et al. (2003) (reported as *Hyla boans*)		LC
Anura	Hylidae	* Boana *	* cinerascens *	X	X	von May et al. (2009)		LC
Anura	Hylidae	* Boana *	*calcarata*	X	X	von May et al. (2009) (reported as *Hypsiboas calcaratus*)		LC
Anura	Hylidae	* Boana *	* geographica *	X	X	Tatum-Hume et al. (2003)(reported as *Hyla geographica*)		LC
Anura	Hylidae	* Boana *	* lanciformis *	X	X	von May et al. (2009), Tatum-Hume et al. (2003) (reported as *Hyla lanciformis*)		LC
Anura	Hylidae	* Boana *	* punctata *	X	X	von May et al. (2009)		LC
Anura	Hylidae	* Boana *	* steinbachi *	X	X	von May et al. (2009) (reported as *Hypsiboas fasciatus*)		NE
Anura	Hylidae	* Callimedusa *	* atelopoides *	X	X	von May et al. (2009), Tatum-Hume et al. (2003) (reported as *Phyllomedusa atelopoides*)		LC
Anura	Hylidae	* Callimedusa *	* tomopterna *	X	X	von May et al. (2009), Tatum-Hume et al. (2003) (reported as *Phyllomedusa tomopterna*)		LC
Anura	Hylidae	* Cruziohyla *	* craspedopus *	X*		Turrell et al. (2016)		LC
Anura	Hylidae	* Dendropsophus *	* acreanus *	X			X	LC
Anura	Hylidae	* Dendropsophus *	* bokermanni *		X		X	LC
Anura	Hylidae	* Dendropsophus *	* brevifrons *	X	X	von May et al. (2009), Tatum-Hume et al. (2003) (reported as *Hyla brevifrons*)		LC
Anura	Hylidae	* Dendropsophus *	* joannae *	X	X		X	LC
Anura	Hylidae	* Dendropsophus *	* kamagarini *	X	X	von May et al. (2009) (reported as *D. parviceps*)		LC
Anura	Hylidae	* Dendropsophus *	* leali *	X	X		X	LC
Anura	Hylidae	* Dendropsophus *	* minutus *	X*		von May et al. (2009)		LC
Anura	Hylidae	* Dendropsophus *	* pauiniensis *		X		X	LC
Anura	Hylidae	* Dendropsophus *	gr. leucophyllatus	X	X	von May et al. (2009) (reported as *D. leucophyllatus*), Tatum-Hume et al. (2003) (reported as *Hyla leucophyllata*)		LC
Anura	Hylidae	* Dendropsophus *	* rhodopeplus *	X	X	von May et al. (2009), Tatum-Hume et al. (2003) (reported as *Hyla rhodopepla*)		LC
Anura	Hylidae	* Dendropsophus *	* salli *	X			X	NE
Anura	Hylidae	* Dendropsophus *	* sarayacuensis *	X	X	von May et al. (2009)		LC
Anura	Hylidae	* Dendropsophus *	* schubarti *		X		X	LC
Anura	Hylidae	* Dendropsophus *	* timbeba *	X				LC
Anura	Hylidae	* Dendropsophus *	* triangulum *	X	X	von May et al. (2009)		LC
Anura	Hylidae	* Osteocephalus *	* castaneicola *	X	X	von May et al. (2009) (reported as *O. leprieurii*)		LC
Anura	Hylidae	* Osteocephalus *	* helenae *	X	X		X	DD
Anura	Hylidae	* Osteocephalus *	* planiceps *		X		X	LC
Anura	Hylidae	* Osteocephalus *	* taurinus *	X	X	von May et al. (2009), Tatum-Hume et al. (2003)		LC
Anura	Hylidae	* Phyllomedusa *	* bicolor *	X	X	von May et al. (2009), Tatum-Hume et al. (2003)		LC
Anura	Hylidae	* Phyllomedusa *	* camba *	X	X	von May et al. (2009), Tatum-Hume et al. (2003)		LC
Anura	Hylidae	* Phyllomedusa *	* palliata *	X	X	von May et al. (2009), Tatum-Hume et al. (2003)		LC
Anura	Hylidae	* Phyllomedusa *	* vaillantii *	X	X	von May et al. (2009)		LC
Anura	Hylidae	* Scarthyla *	* goinorum *	X	X	von May et al. (2009)		LC
Anura	Hylidae	* Scinax *	* garbei *	X	X	von May et al. (2009), Tatum-Hume et al. (2003)		LC
Anura	Hylidae	* Scinax *	* ictericus *	X	X	von May et al. (2009)		LC
Anura	Hylidae	* Scinax *	* pedromedinae *	X	X	von May et al. (2009), Tatum-Hume et al. (2003)		LC
Anura	Hylidae	* Scinax *	* ruber *	X	X	von May et al. (2009), Tatum-Hume et al. (2003) (reported as *Scinax rubra*)		LC
Anura	Hylidae	* Sphaenorhynchus *	* lacteus *	X	X	von May et al. (2009), Tatum-Hume et al. (2003)		LC
Anura	Hylidae	* Trachycephalus *	* coriaceus *	X			X	LC
Anura	Hylidae	* Trachycephalus *	* typhonius *	X	X	von May et al. (2009)		LC
Anura	Leiuperidae	* Edalorhina *	* perezi *	X	X	von May et al. (2009), Tatum-Hume et al. (2003)		LC
Anura	Leptodactylidae	* Adenomera *	aff. hylaedactyla	X	X	von May et al. (2009), Tatum-Hume et al. (2003) (reported as *Adenomera hylaedactyla*)		LC
Anura	Leptodactylidae	* Engystomops *	* petersi *	X	X	von May et al. (2009), Tatum-Hume et al. (2003) (reported as *Physalaemus petersi*)		LC
Anura	Leptodactylidae	* Leptodactylus *	* bolivianus *		X		X	LC
Anura	Leptodactylidae	* Leptodactylus *	* didymus *	X	X	von May et al. (2009)		LC
Anura	Leptodactylidae	* Leptodactylus *	* knudseni *	X	X	von May et al. (2009)		LC
Anura	Leptodactylidae	* Leptodactylus *	* leptodactyloides *	X	X	von May et al. (2009), Tatum-Hume et al. (2003)		LC
Anura	Leptodactylidae	* Leptodactylus *	* pentadactylus *	X	X	von May et al. (2009) , Tatum-Hume et al. (2003)		LC
Anura	Leptodactylidae	* Leptodactylus *	* petersii *	X	X	von May et al. (2009), Tatum-Hume et al. (2003)		LC
Anura	Leptodactylidae	* Leptodactylus *	* rhodomystax *	X		von May et al. (2009)		LC
Anura	Leptodactylidae	* Leptodactylus *	* rhodonotus *		X	von May et al. (2009)		LC
Anura	Leptodactylidae	* Lithodytes *	* lineatus *	X	X	von May et al. (2009)		LC
Anura	Microhylidae	* Chiasmocleis *	* bassleri *	X		von May et al. (2009), Tatum-Hume et al. (2003)		LC
Anura	Microhylidae	* Chiasmocleis *	* ventrimaculata *	X	X	von May et al. (2009) , Tatum-Hume et al. (2003)		LC
Anura	Microhylidae	* Ctenophryne *	* geayi *	X			X	LC
Anura	Microhylidae	* Elachistocleis *	* muiraquitan *	X	X	von May et al. (2009) (reported as *E. bicolor*), Tatum-Hume et al. (2003) (reported as *Elachistocleis bicolor*)		LC
Anura	Microhylidae	* Hamptophryne *	* boliviana *	X	X	von May et al. (2009), Tatum-Hume et al. (2003)		LC
Anura	Pipidae	* Pipa *	* Pipa *	X	X		X	LC
Gymnophiona	Caeciliidae	* Oscaecilia *	* bassleri *				X	LC

**Table 3.** Reptile species found on the Las Piedras River, Madre de Dios, Peru. Most records were documented at Las Piedras Biodiversity Station (LPBS) and the Amazon Research and Conservation Centre (ARCC). * Denotes records not found in our dataset, but confirmed to occur on the Las Piedras Tributary. International Union for Conservation of Nature (IUCN 2025-1) designations denoted as: LC= least risk, LR/cd= least risk/conservation dependent, NE= not evaluated, VU= vulnerable.

**Order**	**Family**	**Genus**	**Specific epithet**	**LPBS**	**ARCC**	**Previously reported on tributary by**	**New records**	**IUCN designation**
Crocodilia	Alligatoridae	* Caiman *	* crocodilus *	X	X	Tatum-Hume et al. (2003)		LC
Crocodilia	Alligatoridae	* Melanosuchus *	* niger *	X	X	Tatum-Hume et al. (2003)		LR/cd
Crocodilia	Alligatoridae	* Paleosuchus *	* palpebrosus *	X	X		X	LC
Crocodilia	Alligatoridae	* Paleosuchus *	* trigonatus *	X	X	Tatum-Hume et al. (2003), Champagne et al. (2015)		LC
Squamata	Alopoglossidae	* Alopoglossus *	* avilapiresae *	X	X		X	NE
Squamata	Amphisbaenidae	* Amphisbaena *	* alba *	X			X	LC
Squamata	Dactyloidae	* Anolis *	* fuscoauratus *	X	X	Tatum-Hume et al. (2003)		LC
Squamata	Dactyloidae	* Anolis *	*nitens*	X*		Tatum-Hume et al. (2003)		
Squamata	Dactyloidae	* Anolis *	*ortoni i*	X*		Tatum-Hume et al. (2003)		
Squamata	Dactyloidae	* Anolis *	* punctatus *	X	X		X	LC
Squamata	Dactyloidae	* Anolis *	* tandai *	X	X		X	LC
*Squamata	Diploglossidae	* Diploglossus *	* fasciatus *	X*		Champagne et al. (2023), Champagne et al. (2024)		
Squamata	Gymnophthalmidae	* Bachia *	* peruana *	X	X		X	LC
Squamata	Gymnophthalmidae	* Cercosaura *	* argulus *	X	X		X	LC
Squamata	Gymnophthalmidae	* Cercosaura *	* eigenmanni *	X		Tatum-Hume et al. (2003)		LC
Squamata	Gymnophthalmidae	* Cercosaura *	* ocellata *	X			X	LC
Squamata	Gymnophthalmidae	* Potamites *	* ecpleopus *	X	X		X	LC
Squamata	Hoplocercidae	* Enyalioides *	* palpebralis *	X	X		X	LC
Squamata	Phyllodactylidae	* Thecadactylus *	* solimoensis *	X	X	Tatum-Hume et al. (2003)(reported as *Thecadactylus rapicauda*)		LC
Squamata	Polychrotidae	* Polychrus *	* liogaster *	X			X	LC
Squamata	Scincidae	* Varzea *	* altamazonica *	X			X	LC
Squamata	Sphaerodactylidae	* Gonatodes *	* hasemanni *	X	X		X	LC
Squamata	Sphaerodactylidae	* Gonatodes *	* humeralis *	X	X	Tatum-Hume et al. (2003)		LC
Squamata	Sphaerodactylidae	* Pseudogonatodes *	* guianensis *	X	X		X	LC
Squamata	Teiidae	* Ameiva *	* ameiva *	X	X	Tatum-Hume et al. (2003)		LC
Squamata	Teiidae	* Dracaena *	* guianensis *				X	LC
Squamata	Teiidae	* Kentropyx *	* altamazonicus *	X	X	Tatum-Hume et al. (2003)		LC
Squamata	Teiidae	* Kentropyx *	* pelviceps *	X	X		X	LC
Squamata	Teiidae	* Tupinambis *	* cuzcoensis *	X	X	Tatum-Hume et al. (2003)(reported as *Tupinambis teguixin*)		NE
Squamata	Tropiduridae	* Plica *	* plica *	X	X	Tatum-Hume et al. (2003)(reported as *Tropidurus plica*)		LC
Squamata	Tropiduridae	* Plica *	* umbra *	X	X		X	LC
Squamata	Tropiduridae	* Stenocercus *	* fimbriatus *	X	X	Tatum-Hume et al. (2003)		LC
Squamata	Tropiduridae	* Stenocercus *	* prionotus *		X		X	LC
Squamata	Tropiduridae	* Stenocercus *	* roseiventris *	X			X	LC
Squamata	Tropiduridae	* Urocentron *	* azureum *		X		X	LC
Squamata	Aniliidae	* Anilius *	* scytale *		X		X	LC
Squamata	Boidae	* Boa *	* constrictor *	X			X	LC
Squamata	Boidae	* Corallus *	* batesii *	X	X		X	LC
Squamata	Boidae	* Corallus *	*hortulana*	X	X	Tatum-Hume et al. (2003)(reported as *Corallus hortulanus*)		LC
Squamata	Boidae	* Epicrates *	* cenchria *	X	X		X	LC
Squamata	Boidae	* Eunectes *	* murinus *	X	X	Champagne (2022), Champagne et al. (2024), Champagne et al. (2025)		LC
Squamata	Colubridae	* Chironius *	* exoletus *	X			X	LC
Squamata	Colubridae	* Chironius *	* fuscus *	X	X		X	LC
Squamata	Colubridae	* Chironius *	* scurrula *	X			X	LC
Squamata	Colubridae	* Chlorosoma *	* viridissimum *				X	LC
Squamata	Colubridae	* Dendrophidion *	* dendrophis *	X	X		X	LC
Squamata	Colubridae	* Drymarchon *	* corais *	X	X	Champagne et al. (2021)		LC
Squamata	Colubridae	* Oxybelis *	* fulgidus *				X	LC
Squamata	Colubridae	* Rhinobothryum *	* lentiginosum *	X	X		X	LC
Squamata	Colubridae	* Spilotes *	* pullatus *	X*		Champagne and Singer (2021)		LC
Squamata	Colubridae	* Tantilla *	* melanocephala *	X	X		X	LC
Squamata	Dipsadidae	* Adelphostigma *	* occipitalis *		X		X	LC
Squamata	Dipsadidae	* Apostolepis *	* nigroterminata *	X			X	LC
Squamata	Dipsadidae	* Atractus *	* emmeli *		X		X	LC
Squamata	Dipsadidae	* Atractus *	* major *		X		X	LC
Squamata	Dipsadidae	* Atractus *	* snethlageae *	X			X	LC
Squamata	Dipsadidae	* Clelia *	* clelia *	X	X	Tatum-Hume et al. (2003), Champagne and Singer (2021), Champagne et al. (2021)		LC
Squamata	Dipsadidae	* Dipsas *	* catesbyi *	X	X	Tatum-Hume et al. (2003)		LC
Squamata	Dipsadidae	* Dipsas *	* indica *	X			X	LC
Squamata	Dipsadidae	* Drepanoides *	* anomalus *	X	X	Crnobrna and Armes (2016)		LC
Squamata	Dipsadidae	* Drymobius *	* rhombifer *		X		X	LC
Squamata	Dipsadidae	* Drymoluber *	* dichrous *	X	X		X	LC
Squamata	Dipsadidae	* Erythrolamprus *	* aesculapii *	X			X	LC
Squamata	Dipsadidae	* Erythrolamprus *	* reginae *	X	X	Tatum-Hume et al. (2003)(reported as *Liophis reginae*)		LC
Squamata	Dipsadidae	* Erythrolamprus *	* taeniogaster *		X		X	LC
Squamata	Dipsadidae	* Helicops *	* angulatus *	X	X	Champagne et al. (2015)		LC
Squamata	Dipsadidae	* Helicops *	* polylepis *	X			X	LC
Squamata	Dipsadidae	* Imantodes *	* cenchoa *	X	X	Tatum-Hume et al. (2003)		LC
Squamata	Dipsadidae	* Imantodes *	* lentiferus *	X			X	LC
Squamata	Dipsadidae	* Leptodeira *	* annulata *	X	X	Tatum-Hume et al. (2003)		LC
Squamata	Dipsadidae	* Leptophis *	* ahaetulla *	X	X		X	LC
Squamata	Dipsadidae	* Oxyrhopus *	* formosus *		X		X	LC
Squamata	Dipsadidae	* Oxyrhopus *	* melanogenys *	X	X	Champagne et al. (2021)		LC
Squamata	Dipsadidae	* Oxyrhopus *	* petolarius *	X			X	LC
Squamata	Dipsadidae	* Phrynonax *	* poecilonotus *	X			X	LC
Squamata	Dipsadidae	* Pseudoboa *	* coronata *	X	X		X	LC
Squamata	Dipsadidae	* Siphlophis *	* cervinus *		X		X	LC
Squamata	Dipsadidae	* Siphlophis *	* compressus *	X	X	Tatum-Hume et al. (2003)(reported as *Tripanurgos compressus*)		LC
Squamata	Dipsadidae	* Xenodon *	* severus *		X		X	LC
Squamata	Dipsadidae	* Xenopholis *	* scalaris *	X	X	Tatum-Hume et al. (2003)		LC
Squamata	Dipsadidae	* Xenoxybelis *	* boulengeri *	X	X		X	LC
Squamata	Elapidae	* Micrurus *	* annelatus *	X	X		X	LC
Squamata	Elapidae	* Micrurus *	* hemprichii *		X		X	LC
Squamata	Elapidae	* Micrurus *	* lemniscatus *	X	X		X	LC
Squamata	Elapidae	* Micrurus *	* obscurus *	X			X	LC
Squamata	Elapidae	* Micrurus *	* surinamensis *	X	X		X	LC
Squamata	Typhlopidae	* Amerotyphlops *	* reticulatus *		X		X	LC
Squamata	Viperidae	* Bothrops *	* atrox *	X	X		X	LC
Squamata	Viperidae	* Bothrops *	* bilineatus *	X	X		X	LC
Squamata	Viperidae	* Lachesis *	* muta *	X	X	Tatum-Hume et al. (2003)		LC
Testudines	Chelidae	* Mesoclemmys *	* gibba *		X		X	NE
Testudines	Chelidae	* Phrynops *	* geoffroanus *	X*			X	NE
Testudines	Chelidae	* Platemys *	* platycephala *		X		X	LC
Testudines	Kinosternidae	* Kinosternon *	* scorpioides *	X			X	LC
Testudines	Pelomedusidae	* Podocnemis *	* unifilis *	X*	X*	Tatum-Hume et al. (2003)		VU
Testudines	Testudinidae	* Chelonoidis *	* denticulatus *	X*	X*	Tatum-Hume et al. (2003)(reported as *Geochelone denticulada*)		VU
